# Neuroimaging in rheumatic diseases

**DOI:** 10.1590/0100-3984.2018.51.4e2

**Published:** 2018

**Authors:** Alair Sarmet Santos

**Affiliations:** 1 Associate Professor in the Department of Radiology of the Fluminense Federal University School of Medicine, Head of the Radiology Department of the Antônio Pedro University Hospital, Niterói, RJ, Brazil

According to the American College of Rheumatology, although rheumatic diseases are rare, one in four American adults has been diagnosed with any of the various types of such diseases. The Centers for Disease Control recently predicted that by the year 2040 approximately 78 million American adults (26% of the population) could be diagnosed with rheumatic disease. Among the rheumatic diseases with central nervous system manifestations, rheumatoid arthritis currently affects about 1.3 million adult Americans and systemic lupus erythematosus (SLE) affects 200,000-300,000^(1)^.

Neuropsychiatric disorders are important clinical manifestations associated with SLE, and the risk of cerebrovascular event is increasing, one of the most serious complications being ischemic stroke, the incidence of which ranges from 3% to 20% in the first five years after the diagnosis of SLE^(2)^. Neurological impairment has a major impact on quality of life and outcomes in patients with SLE, neuroimaging-typically with computed tomography (CT) and magnetic resonance imaging (MRI)-being of great importance for the diagnosis and monitoring of ischemic vascular events^(3,4)^. Advances in MRI and the development of various new related techniques have increased the accuracy of assessments of brain structure, perfusion, and metabolism, thus facilitating the detection and monitoring of brain lesions in such patients^(5)^.

Rheumatic diseases present diverse neurological clinical conditions, and reversible posterior encephalopathy can occur in patients with SLE, especially in women, the main risk factors being active disease, renal impairment, and systemic arterial hypertension. The most common clinical manifestations are seizures, headache, changes in mental state, cortical blindness, clinical signs of hypertension, vomiting, and neurological deficits. Neuroimaging studies show bilateral involvement, most commonly in the occipital and parietal lobes, requiring monitoring of blood pressure and seizures, and reviews of the literature have shown that immunosuppressive drugs produce satisfactory therapeutic results^(6,7)^.

Rheumatoid arthritis is a chronic multisystem inflammatory disease with intra-articular and extra-articular manifestations. Craniovertebral dislocations occur in more than 40% of patients with severe rheumatoid arthritis, and its appropriate diagnosis is fundamental because of the significance of the potential neurological complications. Neuroimaging is essential to confirm the diagnosis and to categorize the severity of the injury. When conservative methods such as traction and external immobilization do not resolve the symptoms or are not indicated, surgical treatment can be necessary. Although intracranial manifestations of rheumatoid arthritis are rare, leptomeningitis can occur, MRI evaluation of the brain and histological analysis of a biopsy specimen being fundamental for diagnostic confirmation^(8,9)^.

Involvement of the central nervous system in patients with scleroderma, which was considered rare, has been reported with increasing frequency. Seizures, headache, involvement of the cranial nerves, hemiparesis, cognitive disorders, and neuropsychiatric disorders have been reported in such patients. In the localized form of scleroderma, the "en coup de sabre" lesion has been described in the frontoparietal region, and the atrophy of muscle structures, cartilage, and facial bones suggest the possibility of Parry-Romberg syndrome. Cerebral atrophy, intraparenchymal calcifications, and white matter lesions, as identified on MRI scans, have also been described. Involvement of the peripheral nervous system (with myopathy, trigeminal neuropathy, and peripheral polyneuropathy) and autonomic nervous system (affecting the cardiovascular and digestive systems) predominate in systemic scleroderma. The treatment of central nervous system involvement varies on a case-by-case basis, diagnostic suspicion and the use of imaging methods being fundamental^(10,11)^.

Behçet's disease is another chronic multisystem inflammatory disorder, and the neurological impairment (referred to as neuro-Behçet's disease) manifests clinically as headache, dysarthria, ataxia, and hemiparesis. Most patients with neuro-Behçet's disease present parenchymal involvement affecting the diencephalic region. There is also an extraparenchymal form, with thrombosis of the cerebral venous sinuses and involvement of the spinal cord. Cerebral venous thrombosis manifests in these patients with neuro-Behçet, with intracranial hypertension syndrome, with the sites most affected being the superior and transverse sagittal sinuses^(12,13)^.

Ankylosing spondylitis is an inflammatory rheumatic disease that mainly affects the axial skeleton. As the disease develops, the spine tends to become rigid, evolving to secondary osteoporosis, increasing the risk of fractures, even with light trauma, and patients can develop a corresponding spinal cord lesion^(14)^. In patients with ankylosing spondylitis who have experienced trauma, evaluation with CT or MRI of the entire spine is recommended, even if the symptoms are mild.

In this issue of **Radiologia Brasileira**, the article "Spectrum of central nervous system involvement in rheumatic diseases: pictorial essay"^(15)^ presents a good review of the main findings of rheumatic diseases and presents cases from two tertiary-care hospitals, with the main neuroradiological findings on MRI and CT, helping alert radiologists to the importance of always investigating the clinical indications and underlying diseases of the patients they treat. A lack of knowledge of the fact that these systemic diseases present neurological manifestations can cause confusion on the part of the radiologist or the attending physician, thus delaying or even impeding the institution of the most appropriate treatment.
